# Complete Genome Sequence of *Lactococcus lactis* subsp. *lactis* A12, a Strain Isolated from Wheat Sourdough

**DOI:** 10.1128/genomeA.00692-16

**Published:** 2016-09-15

**Authors:** Maéva Guellerin, Delphine Passerini, Catherine Fontagné-Faucher, Hervé Robert, Valérie Gabriel, Valentin Loux, Christophe Klopp, Yves Le Loir, Michèle Coddeville, Marie-Line Daveran-Mingot, Paul Ritzenthaler, Pascal Le Bourgeois

**Affiliations:** a Université de Toulouse, Université Paul Sabatier, Toulouse, France; b CNRS, UMR 5100, Toulouse, France; c Université de Toulouse, INSA, UPS, INP, Toulouse, France; d INRA, UMR792, Ingénierie des Systèmes Biologiques et des Procédés, Toulouse, France; e CNRS, UMR5504, Toulouse, France; f Université de Toulouse, Laboratoire de Biotechnologies Agroalimentaire et Environnementale, EA 4565, Institut Universitaire de Technologie, Université Paul Sabatier, Auch, France; g INRA, UR 1077 Mathématique, Informatique et Génome, Jouy-en-Josas, France; h Plateforme Bioinformatique Toulouse, Midi-Pyrénées UBIA, INRA, Auzeville Castanet-Tolosan, France; i INRA, UMR1253 Science et Technologie du Lait et de l'Œuf, Rennes, France; j AGROCAMPUS OUEST, UMR1253 Science et Technologie du Lait et de l’Œuf, Rennes, France

## Abstract

We report here the complete genome sequence of *Lactococcus lactis* subsp. *lactis* strain A12, a strain isolated from sourdough. The circular chromosome and the four plasmids reveal genes involved in carbohydrate metabolism that are potentially required for the persistence of this strain in such a complex ecosystem.

## GENOME ANNOUNCEMENT

Although *Lactococcus lactis* is mostly studied for its role in fermented milk production, it can inhabit nondairy biotopes, such as plant and animal material (1), and is occasionally found among sourdough microflora. *Lactococcus lactis* subsp. *lactis* strain LBAE-A12, abbreviated as strain A12, has been isolated from a French traditional wheat sourdough (2) and belongs to the multilocus sequence type (MLST) group of environmental strains (3). A draft genome sequence of this strain has previously been released (accession no. CBLU000000000.1) for a study combining genomic, transcriptomic, and phenotypic analyses (4).

The complete genome sequence of strain A12 was obtained as follows: a 3-kb-long paired-end library of total DNA was constructed and sequenced on the 454-GS FLX platform (Eurofins MWG Operon), generating 192,126 reads. These reads were used in combination with the 228,613 reads previously obtained (4) from shotgun 454-GS FLX sequencing for the assembly step using the integrated processing environment NG6 (5). A total of 10 scaffolds were generated, and five scaffolds could be ordered and oriented against the high-resolution NheI optical map of the chromosome (6). Chromosomal sequences corresponding to each ribosomal operon (six), gaps (five), nucleotide ambiguities (19 regions), and pseudogenes (78 putative) were determined by Sanger sequencing of appropriate PCR products. Due to the presence of a large DNA duplication on two plasmids, accurate plasmid assembly could be performed only by a combination of numerous methods, such as comparison of 454-generated scaffolds against PacBio RS single-molecule real-time (SMRT) data (39,739 reads), PCR analyses, and Southern hybridization of pulsed-field gel electrophoresis (PFGE) of plasmid linearized by S1 nuclease (7). Annotation of the assembled chromosome and plasmids was performed using the integrated bacterial genome annotation platform AGMIAL (8), followed by a manual curation process.

The complete genome of *L. lactis* subsp. *lactis* A12 consists of a single circular chromosome of 2,603,898 bp (average 35.6% G+C content) containing 2,624 coding sequences (CDSs), including 39 pseudogenes, 65 tRNA genes, and six ribosomal operons. The strain contains four plasmids: pA12-1 (5,736 bp, 33.7% G+C content), pA12-2 (9,105 bp, 34.8% G+C content), pA12-3 (42,401 bp, 33.5% G+C content), and pA12-4 (69,485 bp, 32.9% G+C content). Unexpectedly, the last two plasmids harbor a nearly identical region of 21,475 bp in size that contain genes previously found to be involved in raffinose metabolism (4).

### Accession number(s).

The complete chromosome and plasmid complement of *L. lactis* subsp. *lactis* A12 strain have been deposited in European Nucleotide Archive under the accession numbers LT599049 to LT599053.
